# LncRNA SPOCD1-AS from ovarian cancer extracellular vesicles remodels mesothelial cells to promote peritoneal metastasis via interacting with G3BP1

**DOI:** 10.1186/s13046-021-01899-6

**Published:** 2021-03-16

**Authors:** Conghui Wang, Jiaying Wang, Xiameng Shen, Mingyue Li, Yongfang Yue, Xiaodong Cheng, Weiguo Lu, Xinyu Wang, Xing Xie

**Affiliations:** 1 Women’s Reproductive Health Laboratory of Zhejiang Province, Women’s Hospital, School of Medicine, Zhejiang University, Hangzhou, 310006 Zhejiang China; 2 Department of Gynecologic Oncology, Women’s Hospital, School of Medicine, Zhejiang University, Hangzhou, 310006 Zhejiang China

**Keywords:** Peritoneal metastasis, Mesothelial-to-mesenchymal transition, Extracellular vesicles, SPOCD1-AS, G3BP1, Interfering peptides, Ovarian cancer

## Abstract

**Background:**

Metastasis is the key cause of death in ovarian cancer patients. To figure out the biological nature of cancer metastasis is essential for developing effective targeted therapy. Here we investigate how long non-coding RNA (lncRNA) SPOCD1-AS from ovarian cancer extracellular vesicles (EVs) remodel mesothelial cells through a mesothelial-to-mesenchymal transition (MMT) manner and facilitate peritoneal metastasis.

**Methods:**

EVs purified from ovarian cancer cells and ascites of patients were applied to mesothelial cells. The MMT process of mesothelial cells was assessed by morphology observation, western blot analysis, migration assay and adhesion assay. Altered lncRNAs of EV-treated mesothelial cells were screened by RNA sequencing and identified by qRT-PCR. SPOCD1-AS was overexpressed or silenced by overexpression lentivirus or shRNA, respectively. RNA pull-down and RNA immunoprecipitation assays were conducted to reveal the mechanism by which SPOCD1-AS remodeled mesothelial cells. Interfering peptides were synthesized and applied. Ovarian cancer orthotopic implantation mouse model was established in vivo.

**Results:**

We found that ovarian cancer-secreted EVs could be taken into recipient mesothelial cells, induce the MMT phenotype and enhance cancer cell adhesion to mesothelial cells. Furthermore, SPOCD1-AS embedded in ovarian cancer-secreted EVs was transmitted to mesothelial cells to induce the MMT process and facilitate peritoneal colonization in vitro and in vivo. SPOCD1-AS induced the MMT process of mesothelial cells via interacting with G3BP1 protein. Additionally, G3BP1 interfering peptide based on the F380/F382 residues was able to block SPOCD1-AS/G3BP1 interaction, inhibit the MMT phenotype of mesothelial cells, and diminish peritoneal metastasis in vivo.

**Conclusions:**

Our findings elucidate the mechanism associated with EVs and their cargos in ovarian cancer peritoneal metastasis and may provide a potential approach for metastatic ovarian cancer therapeutics.

**Supplementary Information:**

The online version contains supplementary material available at 10.1186/s13046-021-01899-6.

## Background

Cancer metastasis is the key cause of death in cancer patients. Some of intraperitoneal cancers, such as ovarian cancer and gastrointestinal cancer, frequently spread and colonize on the peritoneum by “seeding” cancer cells along with ascites flowing in the abdominal cavity [1, 2]. A monolayer of mesothelial cells covers the peritoneal surface and acts as the first barrier of cancer cell intraperitoneal implantation. In certain circumstances, such as inflammatory or injury stimulation, mesothelial cells may undergo a mesothelial-mesenchymal transition (MMT) process to obtain a myofibroblast-like phenotype, leading to tissue fibrosis and peritoneal adhesions [3–5]. Recent studies have found that MMT of mesothelial cells also participate in the process of cancer cells adhesion to and colonization of the peritoneum [6–8], but the involved mechanisms remain elusive.

Extracellular vesicles (EVs), initially described as microvesicles, originate from endosomes with a size range from 30 to 150 nm in diameter [9, 10]. Evidence shows that EVs are involved in the metastasis of cancer cells and participate in multiple stages of the metastatic cascade [11–13]. EVs secreted by cancer cells can remodel both neighboring and distant cells to optimize a cancer microenvironment which in return facilitates cancer growth, colonization and metastasis [12, 14]. However, whether cancer derived EVs remodel the peritoneal environment via altering the phenotype of mesothelial cells is poorly understood.

It has been known that EVs contain diverse components, such as nucleic acids, proteins and lipids [9, 15]. Bioactive cargos in EVs are transferred from donor cells to recipient cells to alter the phenotype and function of recipient cells [16, 17]. Long non-coding RNAs (lncRNAs), a group of heterogeneous transcripts with a length of more than 200 nucleotides, have been validated to exist in EVs as well [15, 18]. Despite limited protein coding potential, lncRNAs are involved in transcriptional, post-transcriptional and epigenetic modulation of gene expression [19–21]. Studies show that lncRNAs widely regulate tumor proliferation, drug resistance, metabolism and metastasis [21–23]. However, the association between lncRNAs from cancer EVs and MMT of mesothelial cells needs to be clarified.

Ovarian cancer remains the most lethal malignancy in female reproductive system. More than 70% of ovarian cancer patients are diagnosed with advanced stage at the first visit because of a lack of effective early diagnostic methods [24]. The 5-year overall survival rate of advanced ovarian cancer is only 29% [25]. Ovarian cancer cells shed from the primary site and widely disseminate to multiple intraperitoneal organs, resulting in the poor prognosis of patients. Up to date, there has still been no effective treatment strategy to block peritoneal metastasis of ovarian cancer.

Here, we investigated the role of ovarian cancer secreted EVs in remodeling mesothelial cells through a MMT way. Moreover, we identified an uncharacterized lncRNA in ovarian cancer cell-derived EVs to modulate MMT phenotypes of mesothelial cells, thus promoting cancer cell adhesion to mesothelial cells in vitro and facilitating peritoneal metastasis in vivo, and then confirmed a signaling pathway that participated in MMT process induced by the lncRNA and a specific peptide to block the effect of lncRNA. Our findings of the complex interactions mediated by EVs between cancer cells and non-cancer cells in the peritoneal microenvironment elucidate a new mechanism of cancer peritoneal metastasis and may provide a potential approach for metastatic ovarian cancer therapeutics.

## Methods

### Cells and reagents

Human epithelial ovarian cancer cell lines SKOV3 andA2780 were described previously [26], human mesothelial cell line MeT-5A was purchased from ATCC. Human ovarian epithelial cell line IOSE-80 was kindly provided from Prof. Lu Yan, Zhejiang University. SKOV3, A2780 and IOSE-80 cells were respectively cultured in McCoy’s 5A, RPMI 1640 and DEME medium (BasalMedia, China) supplemented with 10% fetal bovine serum (FBS) (Everyday Green, China). MeT-5A cells were cultured in M199 medium (Hyclone, USA) with 10% FBS (Gibco, USA). SKOV3-luc cells were established by transduction of luciferase-expression lentiviral constructs and cultured in McCoy’s 5A completed medium. All cells were maintained at 37 °C, in 5% CO_2_. TRITC Phalloidin and D-luciferin were purchased from Yeasen (China). Recombinant Human TGF-β1 and recombinant human IL-1β were purchased from R&D Systems (USA). GW4869 (N, N′-Bis [4-(4,5-dihydro-1H-imidazol-2-yl)phenyl]-3,3′-p-phenylene-bis-acrylamide dihydrochloride) was purchased from Sigma-Aldrich (USA). Wheat germ agglutinin was purchased from Invitrogen (USA).

### Patient ascites samples

Ascites from ovarian benign tumor patients (*n* = 2, ovarian fibroma and ovarian mucinous cystadenoma) and ovarian cancer patients (*n* = 3, all high-grade serous ovarian cancer) were collected during surgery. Detailed information of the patients was listed in Table S1. After collection, ascites samples were promptly centrifuged at 3000 g for 15 min at 4 °C to remove cells and cell debris and stored at − 80 °C until use. The usage of ascites samples was approved by the ethical committee of the Women’s Hospital, School of Medicine, Zhejiang University (granted number: 20181063) and informed consent was obtained.

### EV purification and treatment in vitro and in vivo

EVs from cells were purified by differential ultracentrifugation. In brief, after reaching 90% confluency, cells were washed with PBS for three times and incubated with conditioned medium containing EV-depleted FBS (FBS was ultracentrifuged at 120,000 g at 4 °C for 16 h to remove EVs) for 48 h. Then collected supernatant was centrifuged at 300 g for 10 min and then at 2000 g for 10 min at 4 °C, followed by centrifugation at 10,000 g for 30 min to remove cells and cell debris. Next the supernatant was ultracentrifuged at 100,000 g for 90 min to pellet EVs. The pellets were resuspended in PBS, ultracentrifuged at 100,000 g for another 90 min and resuspended in PBS. EVs from ascites were precipitated by ExoQuick Exosome Precipitation Kit (SBI, USA) according to instructions. EVs were ready for cell/mouse treatment or RNA/protein extraction after concentration measurement by BCA Protein Assay Kit (Biotime, China). For cell treatment, MeT-5A cells (1 × 10^5) were seeded in 6-well plates, after adherence, 50 μg EVs were applied to MeT-5A cells for 48 h or 72 h unless otherwise stated. For mouse treatment, 20 μg EVs per 100 μl PBS for each mouse were intraperitoneally injected.

### Transmission electron microscopy (TEM) and particle size analysis

The EV samples were observed by TEM JEM-1230 (JEOL, Japan) after negative staining as previously described [27]. Briefly, EV samples were deposited on copper grids for 10 min and washed with PBS. Excess fluid was blotted away by filter paper. Then grids were transferred to uranyl-oxalate solution (PH = 7) for 5 min, air dried for several minutes and observed using the electron microscope at 80 kV. ZETASIZER Nano (Malvern, UK) was used to analyze the size distribution of EVs according to the instructions.

### PKH67-labelled EV transfer

Purified EVs were incubated with PKH67 (Sigma-Aldrich, USA) for 4 min according to recommendations. The labeling reaction was stopped by 0.5% BSA. The mixture was filtrated through a 0.22 μm filter to remove unbound dye. PKH67-labeled EVs were pelleted and resuspended in PBS. MeT-5A cells (2 × 10^4) were seeded in 4-Chamber Glass Bottom Dish (Cellvis, USA), after adherence, 10 μg PKH67-labeled EVs were incubated with MeT-5A cells for 24 h. After being fixed with 4% paraformaldehyde for 10 min, cell skeleton was labeled with TRITC Phalloidin, nuclei were stained with DAPI and the uptake of EVs into MeT-5A cells was visualized by confocal laser scanning microscope.

### Western blot

Proteins extracted from cells or EVs were separated by 10% SurePAGE gels (GenScript, US) and transferred to PVDF membranes (Bio-Rad, USA) using the eBlot L1 protein transfer system (GenScript, USA). Primary and secondary antibodies used were listed in Table S2. Membranes were exposed to ImageQuant LAS 4000 mini (ImageQuant LAS 4000 mini, USA).

### Immunofluorescence assay

MeT-5A cells (2 × 10^4) were seeded in 4-Chamber Glass Bottom Dish (Cellvis, USA), after treatment, cells were fixed with 4% paraformaldehyde for 10 min, permeabilized with 0.1% Triton X-100 for 5 min and blocked with 2% BSA for 30 min. Then cells were incubated with primary antibodies at 4 °C overnight and secondary antibody at room temperature for 1 h and stained with DAPI. A confocal laser scanning microscope was used to observe and take images. Primary and secondary antibodies used were listed in Table S2.

### Migration assay

Migration assay was performed in 24-well transwell units (8.0 μm pore size, BD, USA). Briefly, MeT-5A cells (1 × 10^5 per well) with different treatments were seeded into the upper chamber with serum-free M199 medium. Completed medium with 10% FBS was added to the lower chamber. After 24 h, the transwell inserts were fixed in 10% formaldehyde and stained with 0.5% crystal violet (Sigma-Aldrich, USA). Images were taken in five randomly selected fields and average number of MeT-5A cells per field was calculated.

### Adhesion assay

Previous studies indicated that the ability of cancer cells attachment to mesothelial cells can be assessed by adhesion assay [28]. In general, MeT-5A cells were seeded into 24-well plates to reach confluence. SKOV3 and A2780 cells (5 × 10^4 per well) stained with 2 μmol/L Cell TrackerTM fluorescent probes Red CMTPX (Invitrogen, USA) were added to MeT-5A cells. After incubation for 30 min at 37 °C, the plates were gently washed with PBS to eliminate non-attached cancer cells. Plates were then fixed with 4% paraformaldehyde and nuclei was stained with DAPI. Images were taken in five randomly selected fields, and average number of adherent SKOV3 and A2780 cells per field was calculated.

### RNA sequencing

To carry out lncRNA sequencing, MeT-5A cells (1 × 10^5) were seeded into 6-well plates. After adherence, 50 μg EVs from two cancer cells (SKOV3 cells and A2780 cells) were co-cultured with MeT-5A cells for 48 h, PBS treatment acted as the control group. Then total RNA of MeT-5A cells was extracted by TRIzol Reagent (Invitrogen, USA). The quantity and quality of RNA were assessed by Agilent 2200 (Agilent, USA). Then sequencing was performed with the illumina Hiseq 3000/4000 by RiboBio CO., LTD (China). First, the rRNA was removed using Epicentre Ribo-Zero rRNA Removal Kit (Illumina, USA). RNA was fragmented to a length about 200 nt, reverse transcribed into single-strand cDNA. Then double-strand cDNA was synthesized and purified, the ends were repaired and linker primers were added for PCR amplification and purification. Finally, the libraries were sequenced after quality inspection. The bioinformatic data were analyzed by RiboBio.

### RNA extraction and qRT-PCR analysis

Total RNA of cells and EVs was extracted by TRIzol Reagent. Cytoplasmic and nuclear RNA was separated using PARIS kit (Life Technologies, USA) according to the protocols. RNA was reverse transcribed by PrimeScript RT reagent Kit with gDNA eraser (Takara, Japan). PCR analysis was conducted using TB Green Premix Ex Taq (Takara, Japan) and 7900HT fast real-time PCR system (Life Technologies, USA). Primer sequences are listed in Table S3. The relative mRNA and lncRNA expressions were calculated by the 2^-ΔΔCt^ method normalized to GAPDH or displayed on 2% agarose gels.

### Northern blot

Northern blot was conducted with DIG Northern Starter Kit (Roche, USA) by Sangon Biotech (Shanghai) Co., Ltd. (China). Briefly, DIG-labeled probes were synthesized using PCR DIG Probe Synthesis Kit (Roche, USA), primer sequences for probe preparation were:

GAPDH forward: 5′-ACTTTGGTATCGTGGAAGGACT-3′;

GAPDH reverse: 5′-TGCTGTAGCCAAATTCGTTGT-3′;

SPOCD1-AS forward: 5′-GCCAGGGAGACCATCTTTTGA-3′;

SPOCD1-AS reverse:5′- AGCTAAGCTGAACACAGTTCT-3′.

Total RNA (15 μg) was loaded onto 1% formaldehyde denatured gel electrophoresis, transferred to a Hybond nylon membrane (Amersham Biosciences, Sweden), and fixed at 80 °C for 2 h. The membrane was prehybridized in DIG Easy Hyb solution (Roche, USA) at 50 °C for 2 h and hybridized with DIG-labeled probes at 50 °C overnight. After washing with 2 × SSC at room temperature for 5 min and washing with 0.1 × SSC at 68 °C for 15 min, the membrane was blocked in Blocking solution for 1 h, incubated with antibody solution for 30 min, and detected using X-ray films.

### Rapid amplification of cDNA ends (RACE)

RACE was performed with GeneRacer™ Kit (Invitrogen, USA) according to the instructions. 5′ RACE and 3′ RACE PCR products were separated on 1.5% agarose gels. Gel extraction products were subcloned to pGM-T vector and bi-directionally sequenced. Primers used for nested PCR of RACE are listed as follows:

5′ RACE outer primer: 5′-CCCCTGGTTGGGGAAGGTTCAAAAG-3′;

5′ RACE inner primer: 5′-GTTGGGGGAGCCCTTCTTCTCTG-3′;

3′ RACE outer primer: 5′-CGAGCCGGAAAAGTTCCCGAAGGA-3′;

3′ RACE inner primer: 5′-CGCTTCCAACTCCTTGCGCTAAGTTC-3′.

### Fluorescence in situ hybridization (FISH)

FISH was performed with Cy3-labeled probes using Ribo™ Fluorescent In Situ Hybridization Kit (RiboBio, China) according to the instructions and previously described [29]. In situ hybridization signals were visualized by a confocal microscope. SPOCD1-AS probes, 18S probes and U6 probes were designed and synthesized by Ribo Bio (China).

### Lentiviruses, plasmids, and siRNAs

SPOCD1-AS overexpression lentivirus (Ubi-MCS-SV40-EGFP-IRES-puromycin), SPOCD1-AS shRNA lentiviruses (hU6-MCS-Ubiquitin-EGFP-IRES-puromycin) and lentiviral construct expressing Luciferase (Ubi-MCS-firefly_Luciferase-IRES-Puromycin) were synthesized by Genechem (China) and transduced into cells according to instructions. Flag-tagged full length human G3BP1, G3BP1 RNA recognition motif (RRM) truncation, and the F380L/F382L mutants of G3BP1 constructs were cloned into a Ubi-MCS-3FLAG-CBh-gcGFP-IRES-puromycin vector (Genechem, China). Transfection of plasmids was performed using X-treme GENE HP DNA Transfection Reagent (Roche, China). G3BP1 inhibitory siRNAs and negative controls were synthesized by Genepharma (China). Transfection of siRNAs was performed using DharmaFECT Transfection Reagents (Thermo, USA).

The target sequences of shRNAs and siRNAs were listed in Table S4. The sequences of primers used for plasmid construction were listed in Table S5. All constructs were confirmed by DNA sequencing.

### RNA pull-down

RNA pull-down assays were performed with biotin-labeled RNA probes synthesized by TsingKe (China) using Pierce™ Magnetic RNA-Protein Pull-Down Kit (Thermo Scientific, USA). Sequences of the mixed sense probes used were 5′-GAACUUUCCGGCUCGAAAU-3′, 5′-AACUUAGCGCAAGGAGUUGG-3′, and 5′-AAGCUGAACACAGUUCUUCC-3′, control probes were the antisense form of the sense probes. Briefly, streptavidin magnetic beads were incubated with SPOCD1-AS sense probes and antisense probes at room temperature for 30 min. After washing three times, the beads were mixed with proteins in RNA-Protein binding buffer at 4 °C overnight. Proteins attached to beads were eluted for next mass spectrometry (MS) by Biotree, China or Western blot analysis.

### RNA immunoprecipitation (RIP)

RIP analysis was conducted using EZ-Magna RIP kit (Millipore, USA) according to instructions. Antibody specific for G3BP1 and isotype control antibody (IgG) were used in the experiments. Briefly, protein A/G magnetic beads were incubated with antibodies at room temperature for 30 min. After washing with RIP wash buffer for three times, the beads were incubated with cell lysates at 4 °C overnight. Co-precipitated RNA was extracted and detected by qRT-PCR. The relative expression of SPOCD1-AS was calculated as input%.

### Interfering peptide synthesis and usage

The interfering peptides with FITC at C-terminus and a cell-penetrating peptide (YGRKKRRQRRR) at N-terminus were synthesized by ChinaPeptides (China). Sequence of the interfering peptide was N′-YGRKKRRQRRRINSGGKLPNFGFVVFDDSEPK-C′, sequence of the negative control peptide was N′-YGRKKRRQRRRDVLGEGNLPVNSPDSILGLKK-C′. The peptides were validated by HPLC (purity > 95%) and MS analysis. For cell treatment, peptides with two concentrations (15 μmol/l and 30 μmol/l) were applied to MeT-5A cells. After incubation for 48 h, MeT-5A cells were fixed for peptides uptake observation and were harvested for further RNA pull-down, western blot, migration and adhesion analysis. For in vivo experiments, peptides (3 mg/kg) were intraperitoneally injected every other day for 10 times.

### Biotin-labeled peptide pull-down

N-terminus biotin-labeled peptides were applied in the experiments. Extracted total RNA was incubated with biotin-labeled peptides (15 μmol/l and 30 μmol/l) at 4 °C overnight. Then RNA-peptide mix was incubated with streptavidin magnetic beads at 4 °C for 2 h. After washing for three times, lncRNA attached to the beads was extracted and measured by qRT-PCR. The relative expression of SPOCD1-AS was calculated as input%.

### Animal studies

Animal experiments were approved by Animal Ethical and Welfare Committee of Zhejiang Chinese Medical University (granted number: IACUC-20180604-06). Female CB-17 severe combined immunodeficiency (SCID) mice (Silaike Experiment Animal Co., Ltd., China) aged 4–6 weeks were used. The ovarian cancer orthotopic mouse model was established by injecting SKOV3-luc cells (1 × 10^6 cells in 10 μl PBS) into the left ovary pulled out from the abdominal midline incision under anesthesia by intraperitoneal injection of 0.3% pentobarbital (0.25 ml/10 g). Tumor development and metastasis were assessed once a week using the In Vivo Imaging System (IVIS) Lumina LT system (PerkinElmer, USA) under anesthesia by isoflurane inhalation after intraperitoneal injection of 150 mg/kg D-luciferin. Bioluminescence data were analyzed using Living Image software (PerkinElmer, USA). One week after cell inoculation, bioluminescence intensity was used to randomize mice into groups with control and different treatments to ensure similar bioluminescence levels in every group (week 1). Six weeks after cell inoculation, mice were sacrificed by intravenous injection of pentobarbital (100 mg/kg). To assess the metastatic tumors, the primary tumors were dissected and bioluminescence of tumors in the peritoneal cavity was obtained. Dissected tissues were fixed in 4% paraformaldehyde and embedded in paraffin for H&E staining.

### Statistical analysis

Statistics were conducted with Graphpad Prism 8.0 (GraphPad Software, USA) and SPSS Statistics 20.0 (IBM, USA). Data in accordance with normal distribution were presented as mean ± standard deviation, and student’s t tests were used to analyze the data between two groups. Otherwise, the data were presented as median ± interquartile range and Mann-Whitney tests were used. Differences with *P* < 0.05 was considered statistically significant.

## Results

### 1. Ovarian cancer-secreted EVs induce the MMT process in mesothelial cells

**Fig. 1:**
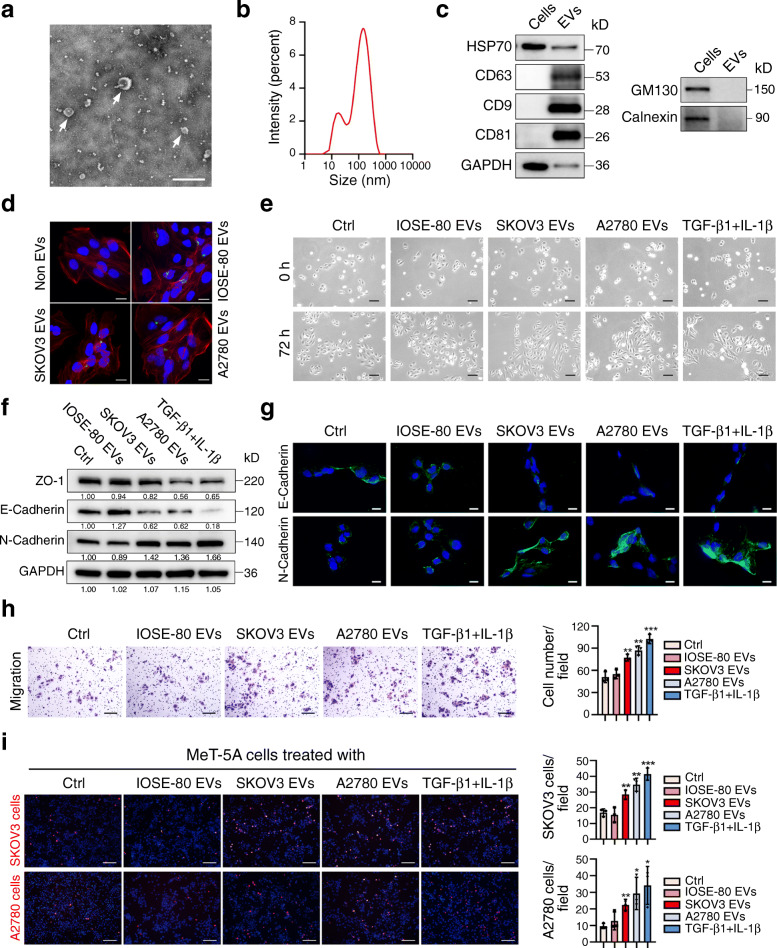
Ovarian cancer-secreted EVs induce the MMT process in mesothelial cells. **a** Representative transmission electron microscopy images of EVs purified from cell supernatant. Scale bar, 200 nm. **b** Size distribution of EVs measured by ZETASIZER Nano. **c** Western blot analysis of HSP70, CD9, CD63, CD81, GAPDH, Calnexin and GM130 in cell lysate and the EV fraction. **d** PKH67-labeled IOSE-80 EVs, SKOV3 EVs and A2780 EVs (green) were incubated with MeT-5A cells for 24 h and observed by confocal microscopy after staining of the F-actin filaments (red) and nuclei (blue), PBS treatment was used as non-EV control. Scale bar, 20 μm. **e** MeT-5A cells were co-cultured with EVs from two cancer cells and IOSE-80 cells. The PBS and TGF-β1 (0.5 ng/ml) plus IL-1β (2.5 ng/ml) groups were used as blank and positive control, respectively. The morphology alteration was observed using a phase contrast microscope. Scale bar, 100 μm. **f** MMT-related proteins of MeT-5A cells with different treatments were detected by western blot, GAPDH was used as the loading control. **g** E-Cadherin and N-Cadherin proteins (green) of MeT-5A cells with different treatments were observed by Immunofluorescence assay. Scale bar, 20 μm. **h** Migration assay was performed to evaluate the migratory ability of MeT-5A cells under different conditions. Representative images were shown and migrated cells were counted. Scale bar, 100 μm. **i** Representative images of CMTPX-labelled SKOV3 (red) and A2780 (red) cells adhesion to MeT-5A cells (blue) were shown and adhered cells were calculated. Scale bar, 100 μm. Data are representative of at least three independent experiments and are presented as mean ± SD. *p < 0.05, **p < 0.01, ***p < 0.001

Two ovarian cancer cell line-secreted EVs (SKOV3 EVs, A2780 EVs) and a normal ovarian epithelial cell line-secreted EVs (IOSE-80 EVs) were purified from cell supernatant. TEM results showed that purified EVs exhibited typical round-shaped morphology with a size of around 100 nm diameter (Fig. 1a). The size of EVs was analyzed by ZETASIZER Nano and mainly ranged from 30 to 100 nm (Fig. 1b). Western blot analysis showed enrichment of positive markers HSP70, CD9, CD63 and CD81, while absence of negative markers Calnexin and GM130 in EVs (Fig. 1c). Labeling and tracking of EVs provide valuable information about secretion, cellular internalization and cargo trafficking of EVs [30]. To investigate whether EVs could transmit between cells, EVs mentioned above were labeled with a widely used lipophilic membrane dye PKH67 and then incubated with peritoneal mesothelial cells (MeT-5A) for 24 h. PKH67 green fluorescence was seen in MeT-5A cells cultured with loaded EVs, but not in control cells (Fig. 1d). To verify whether cancer EVs induce MMT in recipient cells, we treated MeT-5A cells with cancer EVs (SKOV3 EVs and A2780 EVs), IOSE-80 EVs (negative control), and PBS (blank control). The results showed that MeT-5A cells treated with cancer EVs for 72 h acquired an evident spindle-like morphology, similar as cells treated with TGF-β1 plus IL-1β, while the control cells did not show such change (Fig. 1e). The expression of MMT-related genes was detected by Western blot and Immunofluorescence assay in MeT-5A cells treated with cancer EVs for 72 h. ZO-1 and E-Cadherin were repressed, while N-Cadherin was induced, compared to control cells (Fig. 1 f, g). Migration assay showed that more MeT-5A cells migrated when cells were pre-treated with cancer EVs compared to control cells (Fig. 1h). Adhesion assay showed that more SKOV3 or A2780 cells were adhered to MeT-5A cell monolayer pretreated with cancer cell-secreted EVs or TGF-β1 plus IL-1β compared to control cells (Fig. 1i). To further explore whether EVs from ascites of ovarian cancer patients also induce MMT in mesothelial cells, we purified EVs of ascites from three patients with advanced serous ovarian cancer and two other patients with ovarian benign tumor as controls. Patient-secreted EVs also expressed positive EV markers and scarcely expressed Calnexin and GM130 (Fig. S1a). Further, cancer patient-secreted EVs showed the similar abilities to induce MMT of MeT-5A cells, and adhesion of SKOV3 or A2780 cells to MeT-5A cells as cancer cell-secreted EVs (Fig. S1b, c, d, e). Our results suggest that ovarian cancer-secreted EVs possess the ability to remodel the peritoneal mesothelial cells in a MMT manner and enhance cancer cell adhesion to mesothelial cells.

### 2. Screening and identification of lncRNA SPOCD1-AS

**Fig. 2:**
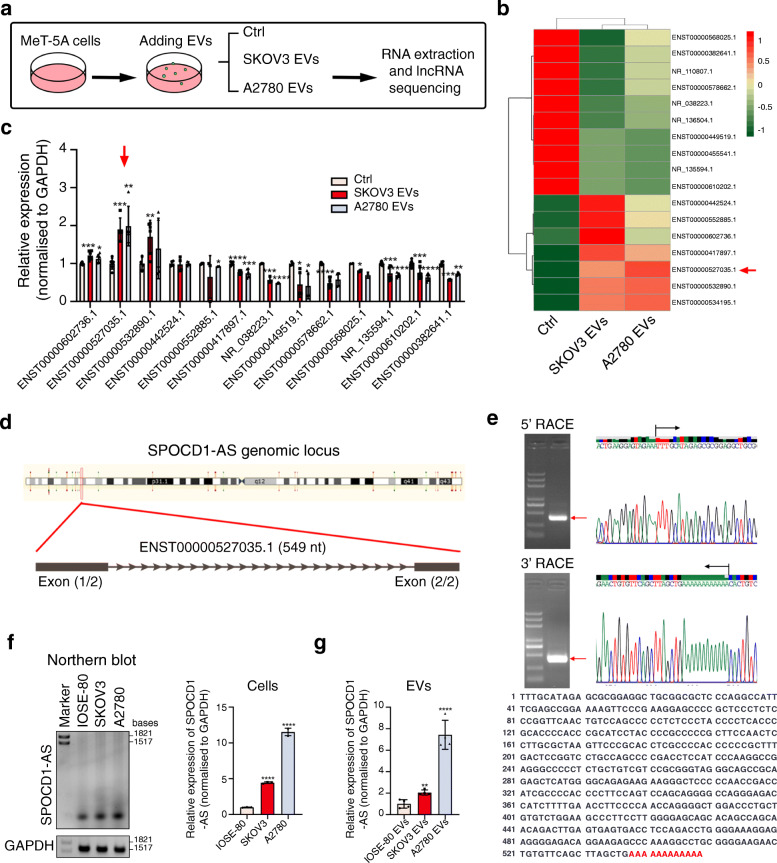
Screening and identification of lncRNA SPOCD1-AS. **a** The schematic protocol of lncRNA expression analysis in MeT-5A cells treated with EVs. **b** The heat map of differentially expressed lncRNAs (cancer EVs vs Ctrl, fold change > 1.5 or < 0.5, *p <* 0.05). The red arrow indicates lncRNA SPOCD1-AS. **c** qRT-PCR analysis of differentially expressed lncRNAs in MeT-5A cells treated with cancer EVs or Ctrl group. Data were normalized to GAPDH and presented as 2^-ΔΔCT^. **d** Schematic annotation of SPOCD1-AS genomic locus on chromosome 1:31,789,130-31,791,322 in human. The black rectangles indicate exons. **e** UP: Agarose gel and sequencing of second-round products of 5′ RACE and 3′ RACE. The red arrows indicate the main PCR products. The vertical line indicates a predicted transcriptional start site or end site. The black arrows indicate transcriptional directions. Down: the full-length nucleotide sequence of SPOCD1-AS. The additional sequence according to Ensembl database reference sequence is marked red. **f** Northern blot using DIG-labeled probes and qRT-PCR analysis of SPOCD1-AS in IOSE-80 and two ovarian cancer cells. GAPDH was used as the loading control. qRT-PCR data were normalized to GAPDH and presented as 2^-ΔΔCT^ compared with IOSE-80 cells. **g** qRT-PCR analysis of SPOCD1-AS in EVs secreted from the three cell lines. Data were normalized to GAPDH and presented as 2^-ΔΔCT^ compared with IOSE-80 EVs. Data are representative of at least three independent experiments and are presented as mean ± SD. *p < 0.05, **p < 0.01, ***p < 0.001, ****p < 0.0001

EVs carry abundant RNA cargos, including miRNAs, mRNAs and long non-coding RNAs, transmit them from donor cells to recipient cells and take effect. Therefore, we performed lncRNA sequencing in MeT-5A cells treated with cancer cell-secreted EVs to determine which lncRNAs were altered. We applied SKOV3 and A2780 EVs to treat MeT-5A cells. After incubation for 48 h, total RNA was extracted and lncRNA sequencing was conducted (Fig. 2a). The heat map showed the differentially expressed lncRNAs between cancer EV-treated and control cells (Fig. 2b). Then we selected top 13 differentially expressed lncRNAs and validated them using qRT-PCR (Fig. 2c). Among these candidate lncRNAs, ENST00000527035.1 was stably and notably up-regulated in MeT-5A cells treated with both SKOV3 and A2780 EVs. We focused on this previously uncharacterized lncRNA and named it as SPOCD1-AS (antisense transcript of SPOCD1 gene). SPOCD1-AS is determined to contain 549 nucleotides by RACE with two exons (Fig. 2 d and e). Coding potential analysis including CPAT [31], CPC2 [32], and PhyloCSF score [33] affirmed that SPOCD1-AS had no coding potential (Table S6). Both Northern blot and qRT-PCR analysis showed the higher expression level of SPOCD1-AS in SKOV3 and A2780 cells than that in IOSE-80 cells (Fig. 2f). Next, we detected SPOCD1-AS in EVs by qRT-PCR and found that cancer EVs contained more SPOCD1-AS than IOSE-80 EVs (Fig. 2g).

### 3. SPOCD1-AS induces the MMT process of mesothelial cells

**Fig. 3:**
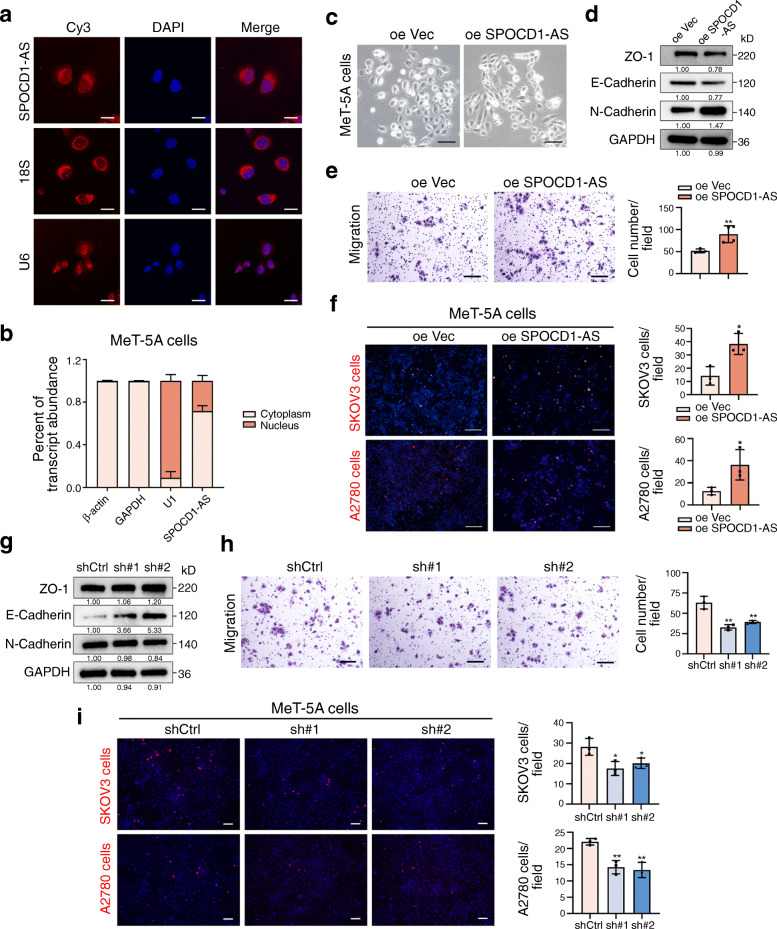
SPOCD1-AS induces the MMT process of mesothelial cells. **a** RNA-FISH analysis of SPOCD1-AS with Cy3-labeled probes (red). 18S and U6 served as the cytoplasm and nucleus control, respectively. Scale bar, 20 μm. **b** qRT-PCR analysis of SPOCD1-AS expressions in cytoplasm and nucleus of MeT-5A cells. GAPDH and β-actin served as the cytoplasm control and U1 as the nucleus control. **c-f** MeT-5A cells were transduced with SPOCD1-AS overexpression and control lentivirus. After transduction, morphology alteration was assessed (**c**), MMT-related proteins were detected by western blot analysis (**d**), migration assay (**e**) and adhesion assay (**f**) were performed. Scale bar, 100 μm. **g-i** MeT-5A cells were transduced with two SPOCD1-AS shRNAs and negative control. After transduction, MMT-related proteins were detected by western blot analysis (**g**), migration and adhesion assays were performed, scale bar, 100 μm (**h**, **i**). Data are representative of at least three independent experiments and are presented as mean ± SD. *p < 0.05, **p < 0.01

We next tried to verify the role of SPOCD1-AS in MeT-5A cells. FISH assay (Fig. 3a) and cell fractionation analysis (Fig. 3b) showed that SPOCD1-AS was mainly localized in cytoplasm. MeT-5A cells with stable overexpression of SPOCD1-AS by lentivirus transfection (Fig. S2a) acquired a spindle-like phenotype (Fig. 3c). Western blot analysis showed that SPOCD1-AS overexpression significantly repressed epithelial-related protein expression and induced mesenchymal-related protein expression (Fig. 3d). Migration assay showed that SPOCD1-AS overexpression promoted the migration in MeT-5A cells (Fig. 3e). Adhesion assay showed that more cancer cells adhered to SPOCD1-AS overexpressed MeT-5A cells (Fig. 3f). Contrarily, MeT-5A cells with stable knockdown of SPOCD1-AS by two shRNAs (Fig. S2b) showed the opposite phenotype (Fig. 3 g, h and i). These results suggest that SPOCD1-AS induces MMT process of peritoneal mesothelial cells and enhances cancer cell adhesion to mesothelial cells.

### 4. EVs carrying SPOCD1-AS induce the MMT process of mesothelial cells

**Fig. 4:**
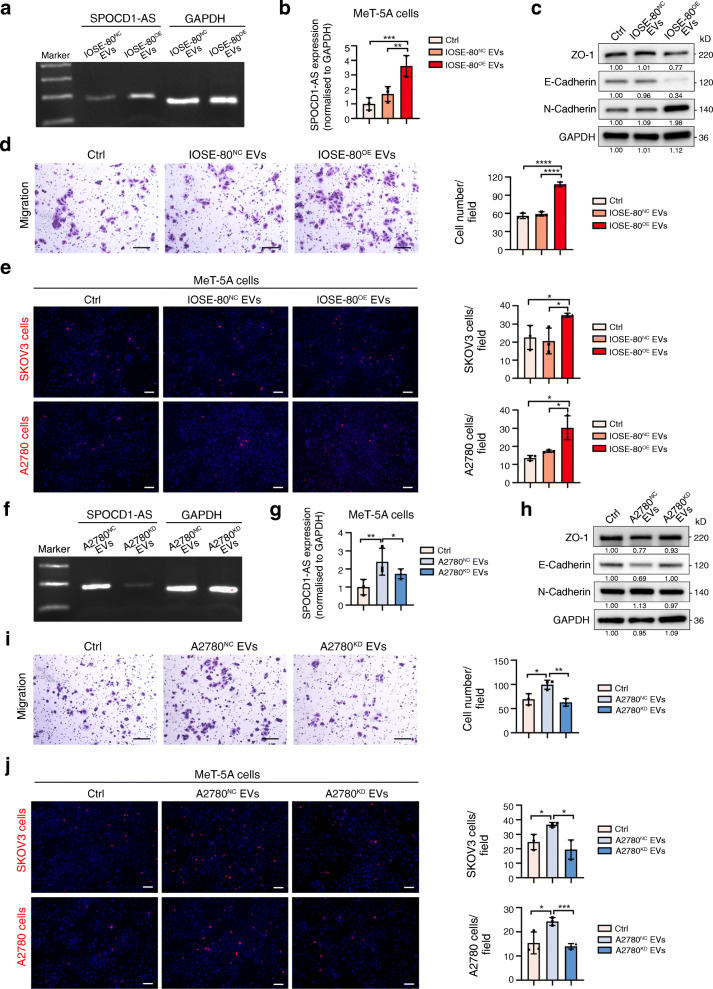
EVs carrying SPOCD1-AS induce the MMT process in mesothelial cells. **a** EVs from control IOSE-80 cells (IOSE-80^NC^ EVs) and IOSE-80 cells with SPOCD1-AS overexpression (IOSE-80^OE^ EVs) were purified. SPOCD1-AS in EVs was detected by qRT-PCR, and PCR product was showed by electrophoresis. **b-e** MeT-5A cells were fed with IOSE-80^NC^ EVs and IOSE-80^OE^ EVs, PBS as control. SPOCD1-AS in MeT-5A cells was detected by qRT-PCR (**b**), MMT-related proteins were detected by western blot analysis (**c**), migration and adhesion assays were performed. Scale bar, 100 μm (**d**, **e**). **f** EVs from control A2780 cells (A2780^NC^ EVs) and A2780 cells with SPOCD1-AS stable knockdown (A2780^KD^ EVs) were purified. SPOCD1-AS in EVs was detected by qRT-PCR, and PCR product was showed by electrophoresis. **g-j** MeT-5A cells were fed with A2780^NC^ EVs, A2780^KD^ EVs, PBS as control. SPOCD1-AS in MeT-5A cells was detected by qRT-PCR (**g**), MMT-related proteins were detected by western blot analysis (**h**), migration and adhesion assays were performed. Scale bar, 100 μm (**i**, **j**). Data are representative of at least three independent experiments and are presented as mean ± SD. *p < 0.05, **p < 0.01, ***p < 0.001, ****p < 0.0001

We further determined whether exogenous SPOCD1-AS from EVs induced MMT in MeT-5A cells. We purified EVs from IOSE-80 cells with SPOCD1-AS stable overexpression (Fig. S3a) (IOSE-80^OE^ EVs) and detected markedly elevated SPOCD1-AS in IOSE-80^OE^ EVs compared with control IOSE-80^NC^ EVs (Fig. 4a). IOSE-80^OE^ EVs increased SPOCD1-AS expression (Fig. 4b), induced MMT traits (Fig. 4 c, d) in MeT-5A cells, and promoted cancer cells adhesion to them (Fig. 4e), compared to EVs from control IOSE-80 cells and blank control. Moreover, we constructed SPOCD1-AS stably silenced A2780 cells (Fig. S3b), whose EVs (A2780^KD^ EVs) scarcely expressed SPOCD1-AS transcripts compared with control A2780^NC^ EVs (Fig. 4f). As expected, A2780^KD^ EVs didn’t exhibit the capacity to elevate SPOCD1-AS level (Fig. 4g) or induce the MMT process of MeT-5A cells (Fig. 4 h, i and j). Thus, these results suggest that excessive SPOCD-AS from cancer cell-secreted EVs is transmitted to recipient mesothelial cells and induces MMT process in the latter.

### 5. EVs carrying SPOCD1-AS promote ovarian cancer peritoneal metastasis in vivo

**Fig. 5:**
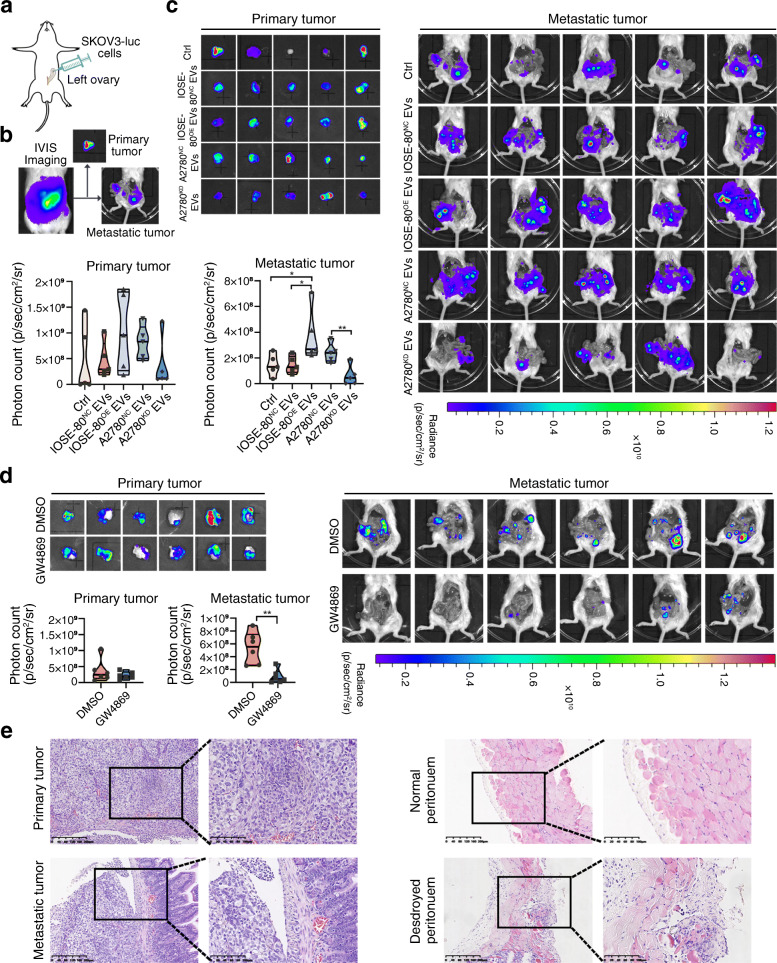
EVs carrying SPOCD1-AS promote ovarian cancer peritoneal metastasis in vivo. **a** Schematic protocol for establishment of orthotopic ovarian cancer mouse model by injecting SKOV3-luc cells (1 × 10^6) into the left ovarian subepithelium. **b** Representative images of orthotopic mouse models at time of killing. The left image indicates the IVIS imaging. The middle image shows dissected primary tumour. The right image showes the metastatic tumours in the peritoneal cavity after the primary tumour was dissected from mice. **c** IOSE-80^NC^ EVs, IOSE-80^OE^ EVs, A2780^NC^ EVs, A2780^KD^ EVs (20 μg per 100 μl for every mouse) and PBS control (100 μl) were intraperitoneally injected every other day from week 1 for 10 times (*n* = 5 for every group). The mice were sacrificed by intravenous injection of pentobarbital (100 mg/kg) at week 6. Bioluminescence images of the dissected primary tumor and the peritoneal metastatic tumors at the time of killing. Photon count was calculated and showed as median with interquartile range. *p < 0.05, **p < 0.01. **d** GW4869 (200 μl of 0.1 mg/ml GW4869 solution, total 20 μg) or 1.25% DMSO saline (200 μl) per mouse were injected intraperitoneal twice a week from week 1 for 8 times (*n* = 6 for each group). Then the mice were sacrificed at week 6. Bioluminescence images of the dissected primary tumor and the peritoneal metastatic tumors at the time of killing. Photon count was calculated and showed as median with interquartile range. **p < 0.01. **e** Histological features of primary and metastatic tumors and peritoneum in mouse model. Hematoxylin and eosin (H&E) staining. Scale bar, 200 μm and 100 μm

We firstly established orthotopic ovarian cancer mouse model to further evaluate the effect of EVs carrying SPOCD1-AS on ovarian cancer peritoneal metastasis in vivo. A median abdominal incision was made and the left ovary was pulled out for each of SCID mice. SKOV3-luc cells (1 × 10^6 cells resuspended in 10 μl PBS) were orthotopically injected into the ovary subepithelium (Fig. 5a). IVIS was conducted every week to monitor tumor progression. Then different EVs (IOSE-80^NC^ EVs, IOSE-80^OE^ EVs, A2780^NC^ EVs, and A2780^KD^ EVs) and PBS control were intraperitoneally injected every other day from week 1 for 10 times. The mice were killed under anesthesia at week 6. To assess the primary and metastasis tumors separately, we conducted IVIS scan of abdomen after taking off the primary tumors (Fig. 5b). Mice injected with IOSE-80^OE^ EVs and A2780^NC^ EVs showed wider peritoneal metastasis compared with those injected with IOSE-80^NC^ EVs, A2780^KD^ EVs or blank control, but there was no difference of primary tumors between EVs with excessive SPOCD-AS and without (Fig. 5c). To further verify the role of EVs on peritoneal metastasis, GW4869, commonly used to inhibit EV generation [34, 35], or DMSO saline was intraperitoneally injected twice a week from week 1 for 8 times, and then the mice were killed under anesthesia. Mice treated with GW4869 showed significant inhibition of peritoneal metastasis, but not primary tumors, compared to control group (Fig. 5d). Histology with HE staining displayed typical morphology of primary and intestinal metastatic adenocarcinoma of ovarian cancer (Fig. 5e, left). We further observed that the mesothelial cell monolayer was intact and connective in normal peritoneum, while the peritoneum with tumor metastasis was destroyed, and sub-mesothelial areas were directly exposed to the peritoneal cavity (Fig. 5e, right).

### 6. SPOCD1-AS induces the MMT process via binding with G3BP1

**Fig. 6:**
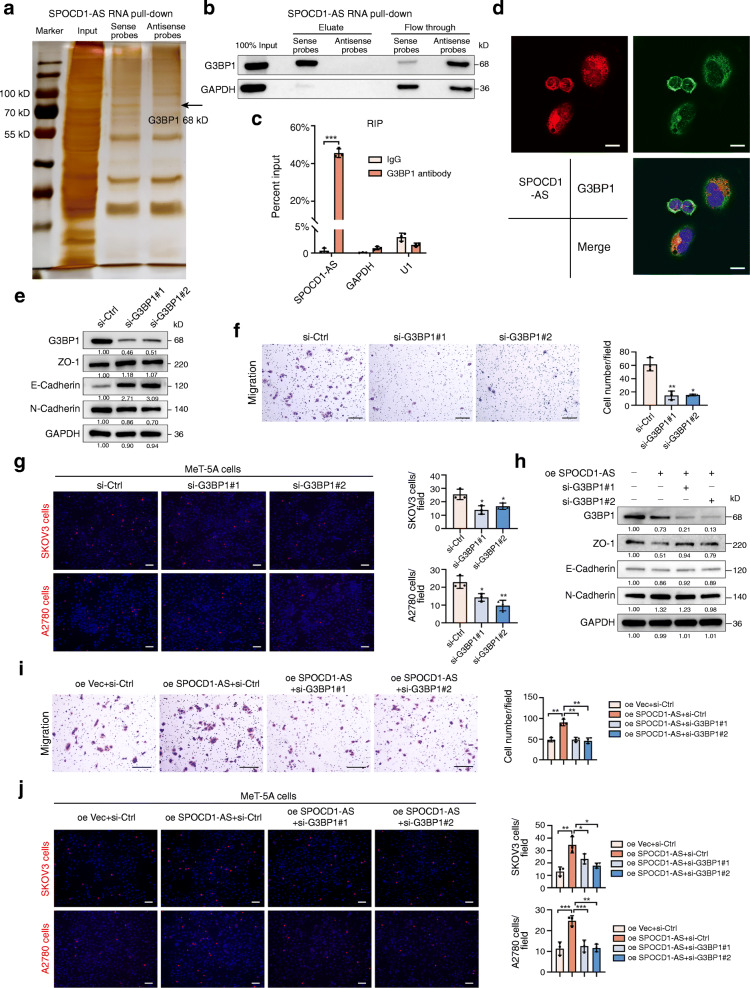
SPOCD1-AS induces the MMT process via binding with G3BP1. **a** RNA pull-down assays in MeT-5A cells using biotin-labeled sense and antisense SPOCD1-AS probes. Sliver staining and mass spectrometry were performed to analyze the proteins pulled down. The black arrow indicates G3BP1 protein band. **b** Western blot validation of protein pulled-down using biotin-labeled sense and antisense SPOCD1-AS probes with G3BP1 antibody. GAPDH was used as the loading control. **c** RNA immunoprecipitation assays in MeT-5A cells using G3BP1 antibody to enrich SPOCD1-AS. GAPDH and U1 were used as negative controls. Data were calculated as input%. **d** RNA-FISH assays of SPOCD1-AS (red) and immunofluorescence of G3BP1 protein (green) in MeT-5A cells. Scale bar, 10 μm. **e, f, g** MeT-5A cells were transfected with two G3BP1 siRNAs and negative control, respectively, for 48 h. MMT-related proteins were detected by western blot analysis (**e**), migration and adhesion assays were performed. Scale bar, 100 μm (**f**, **g**). **h, i, j** MeT-5A cells were transfected with SPOCD1-AS lentivirus, SPOCD1-AS lentivirus plus si-G3BP1#1, SPOCD1-AS lentivirus plus si-G3BP1#2, and negative control, respectively. MMT-related proteins were detected by western blot analysis (**h**), migration and adhesion assays were performed. Scale bar, 100 μm (**i**, **j**). Data are representative of at least three independent experiments and are presented as mean ± SD. *p < 0.05, **p < 0.01, ***p < 0.001

We next explored the underlying mechanism of SPOCD1-AS inducing cellular MMT. It is well understood that lncRNAs drive so many cancer phenotypes through interactions with DNA, RNA or proteins. Therefore, we carried out RNA pull-down assay to find potential proteins interacting with SPOCD1-AS. The proteins pulled down by using biotin-labeled sense probes were displayed by silver staining (Fig. 6a) and then identified by MS (Table S7), antisense probes were used as the negative control. RNA-binding protein Ras-GTPase-activating protein-binding protein 1 (G3BP1) was found to bind to SPOCD1-AS and confirmed by the following Western blot analysis (Fig. 6b). RIP assay was conducted to further verify the interactions between SPOCD1-AS and G3BP1. As shown in Fig. 6c, G3BP1 antibody prominently enriched SPOCD1-AS transcripts in contrast to IgG. RNA-FISH and immunofluorescence assays exhibited that SPOCD-AS and G3BP1 partially co-localized in the cytoplasm of MeT-5A cells (Fig. 6d). Further, downregulation of G3BP1 repressed MMT process of MeT-5A cells (Fig. 6 e and f) and diminished cancer cell adhesion to MeT-5A cells (Fig. 6g). Moreover, SPOCD1-AS overexpression induced MMT and adhesion were partially inhibited by G3BP1 silencing when G3BP1 siRNA was transfected into SPOCD1-AS overexpressed MeT-5A cells (Fig. 6 h, i and j). Our results suggest that SPOCD1-AS induces the MMT process via binding to G3BP1 in MeT-5A cells.

### 7. G3BP1 interfering peptide suppresses the MMT process by blocking SPOCD1-AS/G3BP1 interaction

**Fig. 7:**
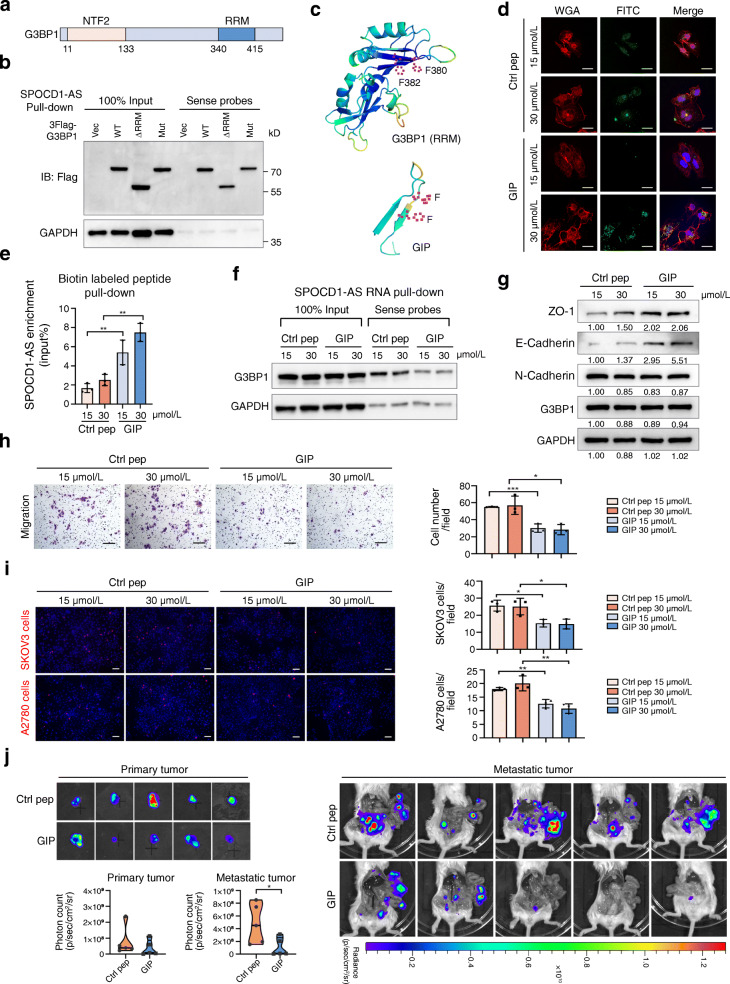
G3BP1 interfering peptide suppresses the MMT process by blocking SPOCD1-AS/G3BP1 interaction. **a** The schematic domain structure of G3BP1. **b** RRM domain truncated and mutant F380L/F382L G3BP1 (marked as ΔRRM and Mut) were detected in RNA pull-down followed with western blot analysis. Empty vector and full-length G3BP1 (marked as Vec and WT) were as controls. **c** The schematic structure of F380 and F382 residues of G3BP1 crucial for SPOCD1-AS/G3BP1 interaction and structure of GIP modeled by Swiss-Model and Pymol. **d** MeT-5A cells were treated with GIP (15 μmol/l and 30 μmol/l) or control peptides (15 μmol/l and 30 μmol/l) for 48 h. Confocal images of green GIP or control peptides in the cytoplasm. Membrane was stained with red wheat germ agglutinin. Scale bar, 20 μm. **e** Biotin-labeled peptide pull-down assays with extracted total RNA of MeT-5A cells. The histogram showed enriched SPOCD1-AS expression, calculated as input%. **f-i** MeT-5A cells were treated with GIP or control peptides for 48 h as mentioned earlier, SPOCD1-AS RNA pull-down assays were conducted with extracted protein of MeT-5A cells using SPOCD1-AS sense probes (**f**), MMT-related proteins were detected by western blot analysis (**g**), migration and adhesion assays were performed. Scale bar, 100 μm (**h**, **i**). **j** Ctrl pep and GIP (3 mg/kg) were intraperitoneal injected every other day from week 1 for 10 times (*n =* 5 for each group). The mice were sacrificed at week 6. Bioluminescence images of the dissected primary tumor and the peritoneal metastatic tumors at the time of killing. Photon count was calculated and showed as median with interquartile range. *p < 0.05. Data are representative of at least three independent experiments and are presented as mean ± SD unless otherwise stated. *p < 0.05, **p < 0.01, ***p < 0.001

Considering that SPOCD1-AS/G3BP1 binding is a key step in inducing MMT process, we further try to block the interaction of SPOCD1-AS and G3BP1. G3BP1 is an RNA-binding protein including an RRM (Fig. 7a), two phenylalanine residues (F380 and F382) were recognized to be crucial for interacting with mRNAs in previous study [36, 37]. Thus, we constructed G3BP1 RRM domain-truncated and F380L/F382L mutant plasmids with 3Flag tag, respectively. After transfecting them into MeT-5A cells, we detected their ability to bind with SPOCD1-AS by RNA pull-down assay followed with Flag protein immunoblotting analysis. The interaction of SPOCD1-AS and G3BP1 with either RRM domain deletion or F380L/F382L mutation was diminished (Fig. 7b). Next, we designed and synthesized a cell-penetrating peptide according to the particular F380 and F382 residues (Fig. 7c) and named it G3BP1 interfering peptide (GIP). GIP was labeled with fluorescein isothiocyanate (FITC) and accessible to the cytoplasm in MeT-5A cells (Fig. 7d). As expected, biotin-labeled peptide pull-down assay showed the binding of GIP to endogenous SPOCD1-AS (Fig. 7e). SPOCD1-AS pull-down assay in MeT-5A cells pre-treated with GIP showed that GIP reduced the endogenous SPOCD1-AS/G3BP1 binding (Fig. 7f), and inhibited the MMT phenotype and cancer cell adhesion to MeT-5A cells (Fig. 7 g, h and i). In orthotopic ovarian cancer mouse models, intraperitoneally injected GIP significantly diminished peritoneal metastasis (Fig. 7j right), but not primary tumors (Fig. 7j left). The results together suggest that GIP we generated possesses the ability to block SPOCD1-AS/G3BP1 interaction, suppress MMT process of MeT-5A cells, and restrains ovarian cancer peritoneal metastasis in vitro and in vivo.

## Discussion

Cancer patients with wide peritoneal dissemination often lose the opportunity to be cured by current treatment methods. Therefore, to explore the biological nature of cancer metastasis and seek new targeted therapy seems extremely urgent. In this study, we uncovered, for the first time to our knowledge, an uncharacterized lncRNA SPOCD1-AS that was secreted from ovarian cancer cells and transmitted to the recipient mesothelial cells through EVs, induced the MMT process in the latter, consequently facilitated peritoneal metastasis. Furthermore, we found that SPOCD1-AS functioned via binding to G3BP1. The blockage of SPOCD1-AS and G3BP1 interaction using a specific interfering peptide GIP attenuated the role of SPOCD1-AS in remodeling mesothelial cells.

EVs have been found to be critical mediators of cell-to-cell communication in recent years [9, 38]. In this study, we initially observed that EVs purified from ovarian cancer cell lines induced mesothelial cells to obtain a pro-metastatic phenotype. Considering both SKOV3 and A2780 cells have some inherent defects, such as flat copy-number profiles, wild-type TP53 and uncharacteristic mutations [39], which may not completely reflect the real situation of ovarian cancer because ovarian cancer in natural state is a group of heterogeneous tumors, we additionally selected EVs from ascites of high-grade serous ovarian cancer patients and still observed the effect of cancer-secreted EVs to induce the MMT process of mesothelial cells. Previous studies showed that EV-transmit miRNAs effectively modulated cell signaling in recipient cells, for instance, breast cancer-secreted miR-105 promoted metastasis [40], and macrophages utilized exosomal miRNAs to regulate insulin sensitivity in adipose tissue [17]. Although much attention has been paid on cargos within EVs in modulating cell-to-cell interactions, little is known about the effect of EVs carrying lncRNAs in remodeling peritoneal mesothelial cells. Accordingly, we screened and identified elevated SPOCD1-AS in cancerous EV-treated mesothelial cells and cancer-secreted EVs. We then found that upregulated SPOCD1-AS induced the MMT features in mesothelial cells, and SPOCD1-AS from ovarian cancer cell-secreted EVs conferred the MMT phenotype of recipient mesothelial cells. We further observed that EVs carrying SPOCD1-AS fostered and GW4869 inhibited cancer peritoneal metastasis in ovarian cancer orthotopic mouse model. Thus, our findings suggest that SPOCD1-AS from ovarian cancer-secreted EVs remodels recipient mesothelial cells in a MMT way, making mesothelial cells in a status conductive to peritoneal implantation of cancer cells.

In general, lncRNA works through target molecules. Previous studies showed that cytoplasm-localized lncRNA-MUF bound to annexin A2 protein to promote hepatocarcinogenesis [41] and lncRNA GLCC1 interacted with protein HSP90 to regulate c-Myc stability [42]. Our FISH assay showed that SPOCD1-AS was mainly located in the cytoplasm, which implied that SPOCD1-AS possibly played a role at the post-transcriptional level. Thus, we utilized RNA pull-down and MS analyses to identify potential proteins interacting with SPOCD1-AS, and focused on G3BP1. Reciprocally, RIP assay indicated the interaction of G3BP1 and SPOCD1-AS. G3BP1 has been reported to be aberrantly expressed and have multiple effects on different cancers. For instance, a study showed that elevated expression of G3BP1 predicted poor prognosis in nonsmall cell lung cancer patients [43]. Our function experiments showed that loss of G3BP1 expression inhibited MMT process and partially neutralized MMT phenotypes induced by SPOCD1-AS in mesothelial cells. Previous studies showed that G3BP1 participated in the epithelial-mesenchymal transition process via modulating Wnt/β-catenin [44], Smad [45], and STAT3 signaling [46] to promote tumor progression and metastasis. Those previous studies support our findings that SPOCD1-AS induces MMT process via binding to G3BP1 in mesothelial cells.

We further verified that RRM domain and F380/F382 residues of G3BP1 were the essential binding sites for SPOCD1-AS and G3BP1 interaction. When full-length G3BP1 plasmid was replaced with G3BP1 with truncated RRM domain or F380L/F382L mutation, the interaction between SPOCD1-AS and G3BP1 was diminished. In recent years, polypeptide drugs are widely applied in different diseases, such as diabetes, obesity, cancers and cardiovascular diseases, owing to their good activity, high specificity and weak immunogenicity [47, 48]. In this study, we generated a cell-penetrating interfering peptide GIP based on the F380/F382 residues, and confirmed its capacity to block the SPOCD1-AS/G3BP1 interaction, inhibit MMT features of mesothelial cells in vitro and suppressed ovarian cancer peritoneal metastasis in vivo. Our findings suggest a potential approach to block intraperitoneal metastasis in ovarian cancer.

## Conclusions

In summary, we demonstrate that SPOCD1-AS from ovarian cancer-secreted EVs remodels mesothelial cells via interacting with G3BP1, and elucidate an unreported process of a crosstalk between cancer cells and mesothelial cells mediated by lncRNA in EVs, accordingly promoting cancer peritoneal metastasis. Our findings may provide a potential approach for metastatic ovarian cancer therapeutics.

## Supplementary Information

**Additional file 1.**

## Data Availability

The datasets used and/or analyzed during the current study are available from the corresponding author on reasonable request.

## Abbreviations

MMT: Mesothelial-to-mesenchymal transition
EVs: Extracellular vesicles
lncRNAs: Long non-coding RNAs
FBS: Fetal bovine serum
TEM: Transmission electron microscopy.
RACE: Rapid amplification of cDNA ends
FISH: Fluorescence in situ hybridization
RRM: RNA recognition motif
MS: Mass spectrometry
RIP: RNA immunoprecipitation
IVIS: In vivo imaging system
SCID: Severe combined immunodeficiency
GIP: G3BP1 interfering peptide
