# Supplementary material for: LncRNA SPOCD1-AS from ovarian cancer extracellular vesicles remodels mesothelial cells to promote peritoneal metastasis via interacting with G3BP1
Source: J Exp Clin Cancer Res. 2021 Mar 16;40:101. doi: 10.1186/s13046-021-01899-6 (PMC7968157; doi:10.1186/s13046-021-01899-6)
Supplement: Supplementary file 1 — Additional file 1. [file 13046_2021_1899_MOESM1_ESM.zip › 2. Supplemental materials.docx]

**Supplemental materials**

**Table S1** Clinical characteristics of ovarian benign tumor and cancer patients

| Patient (ID) | Age | Gender | Pathological diagnosis | FIGO stage | Grade |
| --- | --- | --- | --- | --- | --- |
| B1 | 20 | Female | Ovarian fibroma | N/A | N/A |
| B2 | 54 | Female | Ovarian mucinous cystadenoma | N/A | N/A |
| C1 | 50 | Female | Serous ovarian cancer | III | High grade |
| C2 | 37 | Female | Serous ovarian cancer | III | High grade |
| C3 | 46 | Female | Serous ovarian cancer | III | High grade |

**Table S2** Antibodies used in this study.

| Antigens | Manufacturer | Application |
| --- | --- | --- |
| CD9 | ExoAb Antibody Kit,  SBI, USA | 1:1000 for WB |
| CD63 |  | 1:1000 for WB |
| CD81 |  | 1:1000 for WB |
| HSP70 |  | 1:1000 for WB |
| Calnexin (A4846) | Abclonal, China | 1:1000 for WB |
| GM130 (A18042) | Abclonal, China | 1:1000 for WB |
| ZO-1 (D7D12) | CST, USA | 1:1000 for WB |
| N-Cadherin (D4R1H) | CST, USA | 1:1000 for WB  1:200 for IF |
| E-Cadherin (EP700Y) | Abcam, USA | 1:1000 for WB  1:400 for IF |
| GAPDH | Diagbio, China | 1:1000 for WB |
| G3BP1 (H10) | Santa Cruz, USA | 1:400 for WB, 1:50 for IF, 5 µg per reaction for RIP |
| Flag | Multi Sciences, China | 1:1000 for WB |
| HRP-linked Anti-rabbit IgG | CST, USA | 1:2000 for WB |
| Goat Anti Mouse IgG (H+L)-HRP | Diagbio, China | 1:2000 for WB |
| Mouse anti-rabbit IgG-FITC (sc2359) | Santa Cruz, USA | 1:400 for IF |

**Table S3** Sequences of primers used for qRT-PCR in this study.

| Item | Sequence | |
| --- | --- | --- |
| ENST00000602736.1 | Forward (5’-3’) | GGAACATCTGGAATGCGCTTGC |
|  | Reverse (5’-3’) | GATCATCTCAGTGTGACAGCCTCTTC |
| ENST00000527035.1 | Forward (5’-3’) | GCCAGGGAGACCATCTTTTGA |
|  | Reverse (5’-3’) | AGCTAAGCTGAACACAGTTCT |
| ENST00000532890.1 | Forward (5’-3’) | CGGAGGATTGTGCCTGAGAAGAAC |
|  | Reverse (5’-3’) | ACTTCATAGTTGGCTTGAGAGTCCATC |
| ENST00000442524.1 | Forward (5’-3’) | GATCTGACAGATGCCAGCGTAGC |
|  | Reverse (5’-3’) | CGAGATCACACCACCGCACTTC |
| ENST00000552885.1 | Forward (5’-3’) | GAATGGAATGGAGGATAGGTCGTGAAG |
|  | Reverse (5’-3’) | AGCAATGGAGTCTGAAGCAAGTTAGG |
| ENST00000417897.1 | Forward (5’-3’) | GCCGTCACTTAGTCGCCGATC |
|  | Reverse (5’-3’) | TTATTCAGCATCCGATCCAAGTCCTAC |
| NR_038223.1 | Forward (5’-3’) | CATTCCCAGAAACTTCCAGCAGAGG |
|  | Reverse (5’-3’) | GGCAGGCAGGATAGCATGTTAGC |
| ENST00000449519.1 | Forward (5’-3’) | AAGTCTCATCTGTCACTGTGCTTGC |
|  | Reverse (5’-3’) | CCAGAGGTGAGGAGATAGTAGAGGAAG |
| ENST00000578662.1 | Forward (5’-3’) | AGTGGAGGAAGAGGCATGGAGATC |
|  | Reverse (5’-3’) | CTAGGAGAAGAGCAGAGGAGACAGG |
| ENST00000568025.1 | Forward (5’-3’) | CTGAGGCAGGTGGATCACTTGAAG |
|  | Reverse (5’-3’) | ATGGCACAATCTCGGATCACTACAAC |
| NR_135594.1 | Forward (5’-3’) | CGATATTAGTGTCACCGCCGTCAG |
|  | Reverse (5’-3’) | GCCACCTTGTCTCTGCTTCCAC |
| ENST00000610202.1 | Forward (5’-3’) | CGATATTAGTGTCACCGCCGTCAG |
|  | Reverse (5’-3’) | GCCACCTTGTCTCTGCTTCCAC |
| ENST00000382641.1 | Forward (5’-3’) | GCACAGCACCAGCGGACAG |
|  | Reverse (5’-3’) | CGGACCTGCGTGCGTTCTTC |
| GAPDH | Forward (5’-3’) | TCACCACCATGGAGAAGGC |
|  | Reverse (5’-3’) | GCTAAGCAGTTGGTGGTGCA |
| b-actin | Forward (5’-3’) | AATCGTGCGTGACATTAAGGAG |
|  | Reverse (5’-3’) | ACTGTGTTGGCGTACAGGTCTT |
| U6 | Forward (5’-3’) | CTCGCTTCGGCAGCACA |
|  | RT (5’-3’) | AACGCTTCACGAATTTGCGT |
| U1 | Forward (5’-3’) | CCATGATCACGAAGGTGGTTT |
|  | Reverse (5’-3’) | ATGCAGTCGAGTTTCCCACAT |
| G3BP1 | Forward (5’-3’) | CGGGCGGGAATTTGTGAGA |
|  | Reverse (5’-3’) | TCTGTCCGTAGACTGCATCTG |

**Table S4** Sequences of shRNAs and siRNAs against specific targets in this study.

| Item | Sequence | |
| --- | --- | --- |
| SPOCD1-AS shRNA-1 | Sense (5’-3’) | CTTCCAACTCCTTGCGCTA |
|  | Antisense (5’-3’) | TAGCGCAAGGAGTTGGAAG |
| SPOCD1-AS  shRNA-2 | Sense (5’-3’) | GCGCTAAGTTCCCGCACCT |
|  | Antisense (5’-3’) | AGGTGCGGGAACTTAGCGC |
| G3BP1 siRNA-1 | Sense (5’-3’) | GGGCUUCUCUCUAACAACATT |
|  | Antisense (5’-3’) | UGUUGUUAGAGAGAAGCCCTT |
| G3BP1 siRNA-2 | Sense (5’-3’) | GCGAGAACAACGAAUAAAUTT |
|  | Antisense (5’-3’) | AUUUAUUCGUUGUUCUCGCTT |
| Ctrl siRNA | Sense (5’-3’) | UUCUCCGAACGUGUCACGUTT |
|  | Antisense (5’-3’) | ACGUGACACGUUCGGAGAATT |

**Table S5** Sequences of primers used for plasmid construction in this study.

| Item | Sequence | |
| --- | --- | --- |
| SPOCD1-AS  lentivirus | Forward (5’-3’) | GAGGATCCCCGGGTACCGGTTTTGC  ATAGAGCGCGGAGGCTG |
|  | Reverse (5’-3’) | CACACATTCCACAGGCTAGCAGCTAA  GCTGAACACAGTTC |
| SPOCD1-AS shRNA-1 | Sense (5’-3’) | CCGGCTTCCAACTCCTTGCGCTACTCG  AGTAGCGCAAGGAGTTGGAAGTTTTTG |
|  | Antisense (5’-3’) | AATTCAAAAACTTCCAACTCCTTGCGCT  ACTCGAGTAGCGCAAGGAGTTGGAAG |
| SPOCD1-AS  shRNA-2 | Sense (5’-3’) | CCGGGCGCTAAGTTCCCGCACCTCTCG  AGAGGTGCGGGAACTTAGCGCTTTTTG |
|  | Antisense (5’-3’) | AATTCAAAAAGCGCTAAGTTCCCGCACCT  CTCGAG AGGTGCGGGAACTTAGCGC |
| G3BP1 plasmids | Forward (5’-3’) | AGGTCGACTCTAGAGGATCCCGCCACCAT  GGTGATGGAGAAGCCTAG |
|  | Reverse (5’-3’) | TCCTTGTAGTCCATACCCTGCCGTGGCGC  AAGCCCCCTTC |

**Table S6, related to Figure 2.** Protein coding potential of human lncRNA ENST00000527035.1.

| Metric | Raw result | Interpretation |  |
| --- | --- | --- | --- |
| CPAT | 0.00407 | Non-coding | |
| CPC2 | 0.01593 | Non-coding | |
| PhyloCSF score | Negative value | Non-coding | |

**Table S7, related to Figure 6.** Peptide information of G3BP1 from the mass spectrometry data.

| Protein | Peptide |
| --- | --- |
| G3BP1  [Homo sapiens] | VPASQPRPESKPESQIPPQRPQR |
|  | EAGEQGDIEPR |
|  | HPDSHQLFIGNLPHEVDKSELK |
|  | FYVHNDIFR |
|  | TFSWASVTSK |

**Figure legend**

**Figure S1, related to Figure 1.** (**a**) Western blot analysis of HSP70, CD9, CD63, CD81, GAPDH, Calnexin and GM130 in patient ascites-derived EVs. MeT-5A cells were co-cultured with equal quantities of EVs from ascites (two benign tumor patients: B1, B2, three cancer patients: C1, C2, C3), (**b**) the morphology alteration was observed using a phase contrast microscope, scale bar, 100 μm; (**c**) Western blot analysis of MeT-5A cells under different conditions; (**d**) Migration assay of MeT-5A cells pretreated with different EVs, representative images were shown and migrated cells were counted, scale bar, 100 μm; (**e**) Adhesion assay of SKOV3 (red) and A2780 (red) cells adhered to MeT-5A cells under different conditions, representative images were shown and adhered cells were counted, scale bar, 100 μm.

Data are representative of at least three independent experiments and are presented as mean ± SD. *p<0.05, **p<0.01.

**Figure S2, related to Figure 3.** (**a**) MeT-5A cells were transfected with SPOCD1-AS overexpression and control lentivirus. SPOCD1-AS expression was detected using qRT-PCR analysis. Data were normalized to GAPDH and presented as 2^-ΔΔCT^. (**b**) MeT-5A cells were transfected with two SPOCD1-AS shRNAs and negative control. SPOCD1-AS expression was detected using qRT-PCR analysis. Data were normalized to GAPDH and presented as 2^-ΔΔCT^.

Data are representative of at least three independent experiments and are presented as mean ± SD. ***p<0.001, ****p<0.0001.

**Figure S3, related to Figure 4.** (**a**) IOSE-80 cells were transfected with SPOCD1-AS overexpression and control lentivirus. SPOCD1-AS expression was detected using qRT-PCR analysis. Data were normalized to GAPDH and presented as 2^-ΔΔCT^. (**b**) A2780 cells were transfected with SPOCD1-AS shRNA sh#1 and negative control. SPOCD1-AS expression was detected using qRT-PCR analysis. Data were normalized to GAPDH and presented as 2^-ΔΔCT^.

Data are representative of at least three independent experiments and are presented as mean ± SD. **p<0.01, ***p<0.001.
