# Supplementary figures and images for: LncRNA SPOCD1-AS from ovarian cancer extracellular vesicles remodels mesothelial cells to promote peritoneal metastasis via interacting with G3BP1
Source: J Exp Clin Cancer Res. 2021 Mar 16;40:101. doi: 10.1186/s13046-021-01899-6 (PMC7968157; doi:10.1186/s13046-021-01899-6)

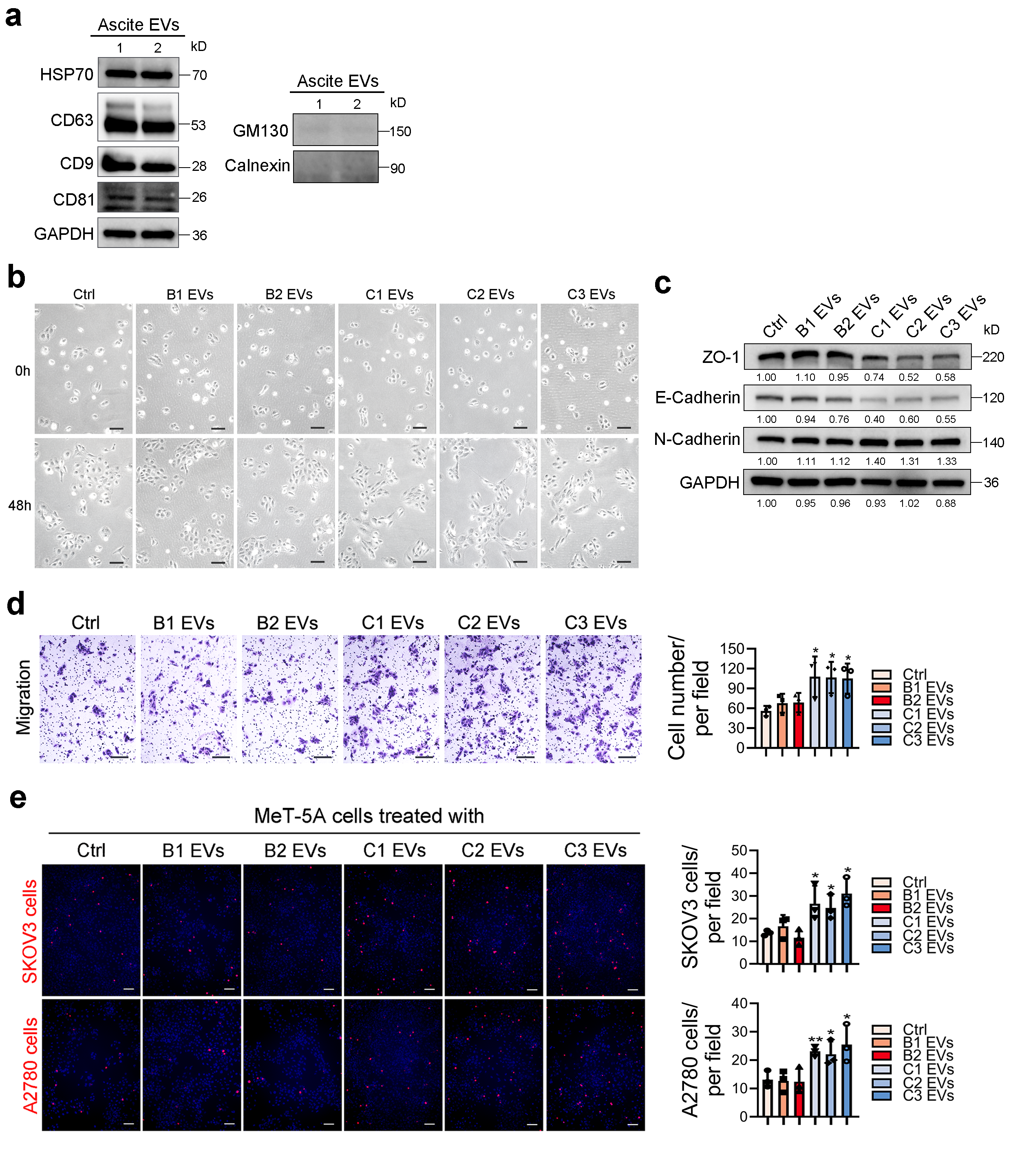

Supplement: Supplementary file 1 — Additional file 1. [file 13046_2021_1899_MOESM1_ESM.zip › FigureS1.tif]

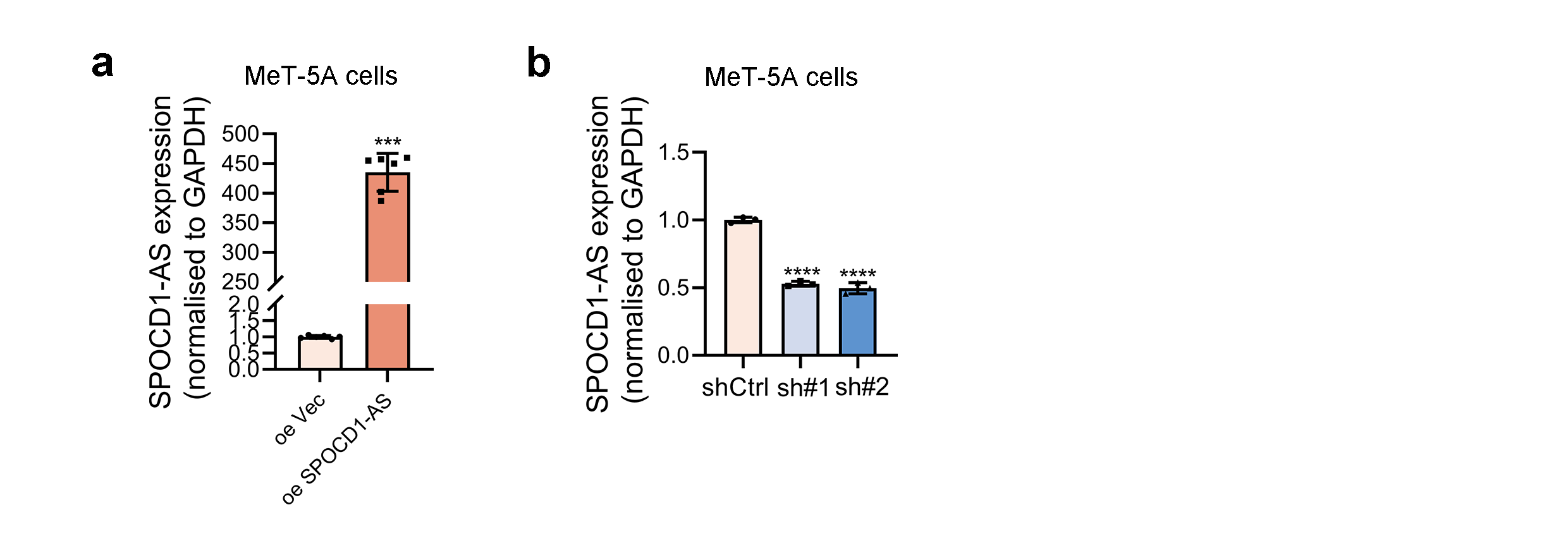

Supplement: Supplementary file 1 — Additional file 1. [file 13046_2021_1899_MOESM1_ESM.zip › FigureS2.tif]

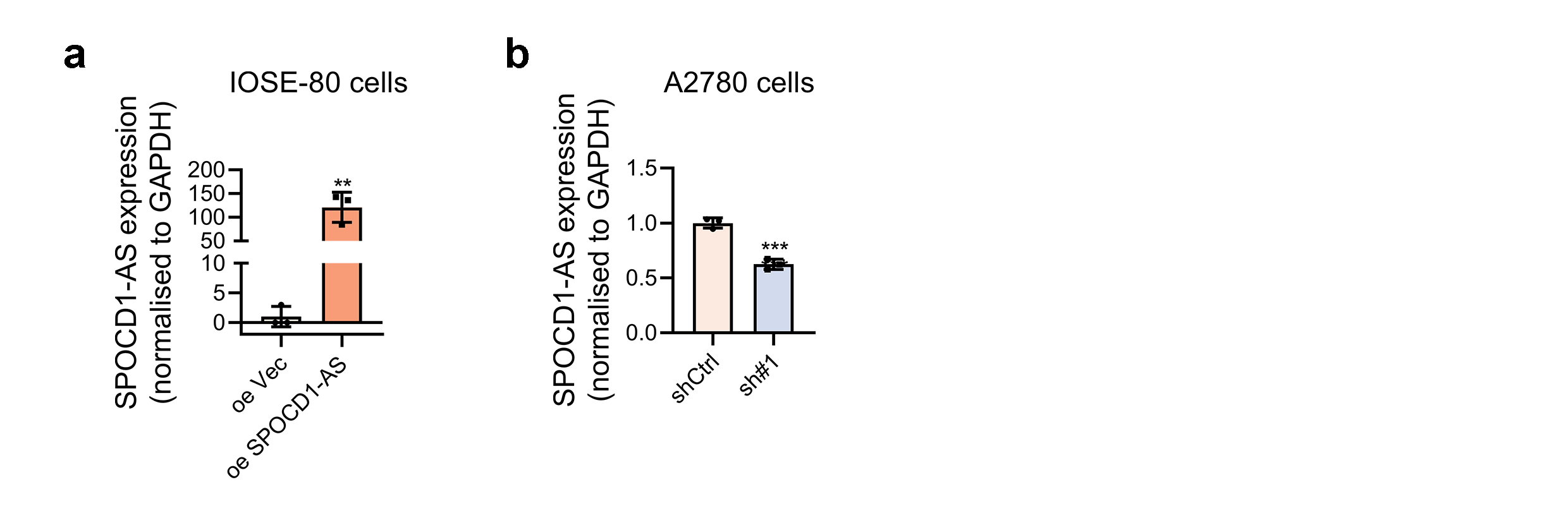

Supplement: Supplementary file 1 — Additional file 1. [file 13046_2021_1899_MOESM1_ESM.zip › FigureS3.tif]
